# Real-world characteristics and outcomes of patients with multiple myeloma treated with belantamab mafodotin: a German claims data study

**DOI:** 10.1007/s00277-025-06427-6

**Published:** 2025-05-31

**Authors:** Jaime Luna, Tim d’Estrube, Andreas Jacobsen, Moritz Lehne, Birgit Wellmann-Pichler, Antje Mevius, Sabine Dornig

**Affiliations:** 1 Cytel Inc, Berlin, Germany; 2 GSK, London, UK; 3 GSK, Munich, Germany; 4 Institut für Pharmakoökonomie und Arzneimittellogistik e.V. (IPAM), Wismar, Germany; 5 AOK PLUS, Dresden, Germany

**Keywords:** Belamaf, Belantamab mafodotin, Ocular events, Real-world evidence, Relapsed/refractory multiple myeloma

## Abstract

In the DREAMM-2 trial, belantamab mafodotin (belamaf) monotherapy demonstrated clinical activity and a manageable safety profile in pretreated patients with relapsed/refractory multiple myeloma (RRMM). Real-world evidence on this patient population is scarce. This study describes real-world baseline characteristics, ocular events, and time-to-event outcomes of patients with RRMM before, during, and after belamaf treatment between August 1, 2020, and March 31, 2023, in Germany based on anonymized health insurance claims data. In the overall cohort (*N* = 31), the median age was 66 years and all patients had received several MM therapies prior to treatment with belamaf. Of the patients included in the pre-belamaf (*n* = 31), belamaf (*n* = 31), and post-belamaf (*n* = 22) treatment periods, 10 (32.3%), 16 (51.6%), and 15 (68.2%) had at least one recorded ophthalmological visit, respectively. Keratitis was the most frequent among ocular diagnoses of interest across all three study periods. During the belamaf treatment period, seven (22.6%) patients had one or more incident ocular diagnoses of interest, the most frequent being corneal-related events. In the overall cohort, the real-world progression-free survival and overall survival were 4.3 months (95% confidence interval [CI] 2.4–7.7) and 12.3 months (95% CI 6.9–not evaluable), respectively. Our data emphasize the importance of ocular supportive care during belamaf treatment in real-world clinical practice. Our findings also highlight that RRMM remains an aggressive disease with a poor prognosis.

## Introduction

Multiple myeloma (MM) is the second most common hematological malignancy in Europe, with an estimated incidence of 4.5–6 cases per 100 000 people per year [1]. In Germany, approximately 3000 women and 3700 men are diagnosed with MM every year [2]. Novel therapies have recently led to improvements in clinical outcomes of patients with MM; however, most patients relapse or become refractory to treatment [3]. In a study across seven European countries, 61% of patients with MM received second-line treatment after relapse, 38% progressed to third-line, 15% progressed to fourth-line, and 1% progressed to fifth-line treatments [4].

In the phase II DREAMM-2 trial, single-agent belantamab mafodotin (belamaf), a B-cell maturation antigen-targeted antibody–drug conjugate, demonstrated clinical activity and a manageable safety profile in pretreated patients with relapsed/refractory MM (RRMM) [5, 6]. Belamaf is currently being investigated for use in combination treatment regimens. The phase III DREAMM-7 and DREAMM-8 clinical trials have recently reported encouraging efficacy outcomes in patients with RRMM treated with belamaf combined with bortezomib–dexamethasone (Vd) and pomalidomide–dexamethasone (Pd), respectively, after first relapse [7, 8]. Belamaf was indicated in Europe (August 2020 until December 2023) as monotherapy for the treatment of MM in adult patients who received at least four prior therapies and whose disease was refractory to at least one proteasome inhibitor, one immunomodulatory agent, and a monoclonal CD38 antibody, and who have demonstrated disease progression on the last therapy [9].

Belamaf has been associated with ocular toxicities due to microtubule-disrupting monomethyl auristatin-F, a cytotoxic payload of the drug that causes off-target damage to the corneal epithelial cells [10]. Of the 218 patients treated with belamaf monotherapy in the DREAMM-2 pooled safety population, ocular adverse events included keratopathy (76%), decreased visual acuity (55%), blurred vision (27%), and dry eye (19%) [11]. Other adverse events reported in DREAMM-2 were eye pruritus, ulcerative keratitis, infective keratitis, ocular discomfort, visual impairment, photophobia, and blepharitis [6]. The original European label recommended ophthalmic examinations before belamaf treatment to set a baseline over the initial period of treatment to monitor potential changes to the patient’s eyes [9].

Due to the relatively recent introduction of belamaf to the RRMM therapeutic landscape in August 2020, real-world evidence on patients treated with belamaf is scarce. This study aimed to describe the baseline characteristics of patients with RRMM treated with belamaf in Germany. Additionally, the study aimed to describe the documented ocular events from an insurance claims perspective, and time-to-event outcomes for this patient population in a real-world setting. Obtaining real-world evidence data will be beneficial for patients with RRMM, healthcare professionals, and payers.

## Methods

### Study design and patient population

**Fig. 1:**
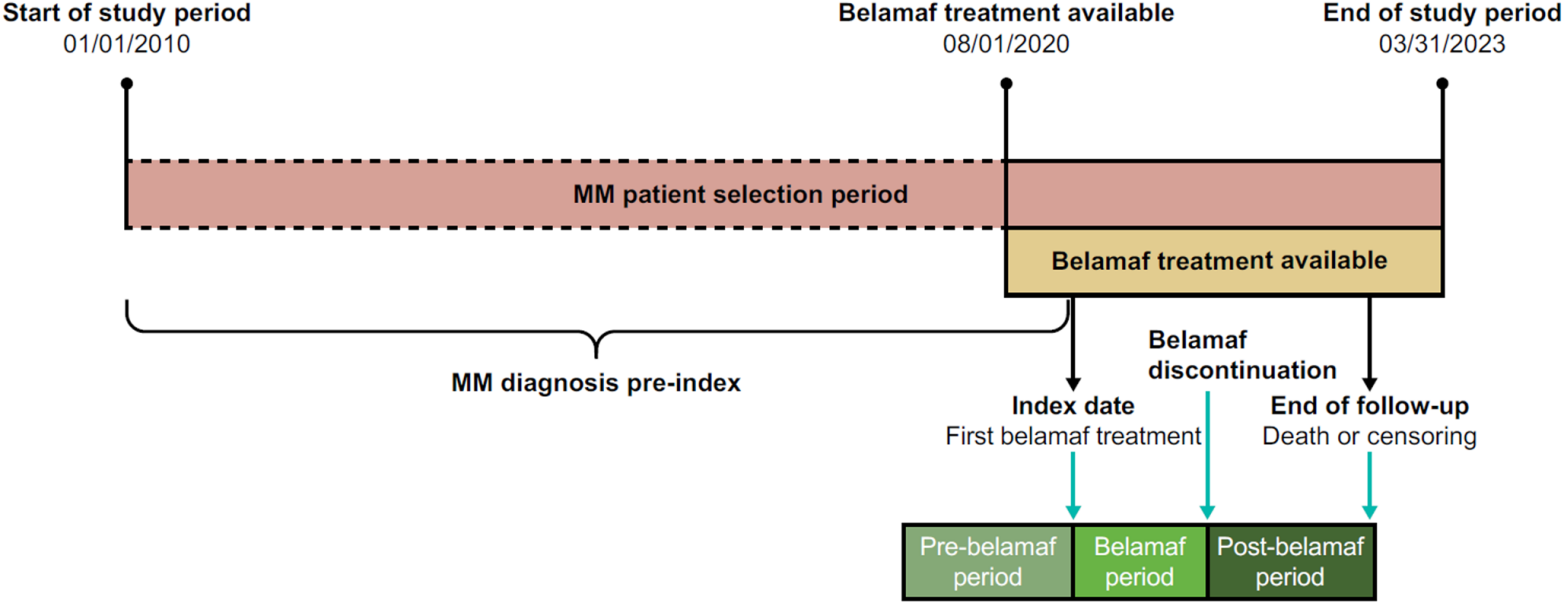
Study design. Patients with a belamaf treatment between August 1, 2020, and March 31, 2023 (latest data available), and diagnosed with MM between January 1, 2010, and first belamaf treatment, were identified. Primary outcomes were investigated at index (first belamaf treatment) or in the 12-month baseline period relative to the first belamaf treatment (baseline characteristics); secondary outcomes investigated pre-, during, and post-belamaf treatment. The index date as well as the pre-belamaf, belamaf, and post-belamaf periods are illustrated based on a hypothetical patient with that specific index date. The pre-belamaf period was defined as the time from 1 month before the start of belamaf treatment to the start of belamaf treatment. The belamaf period started at the index date and ended on the last day of the first instance of a 90-day period without a subsequent dose, the end of follow-up, or the date of death, whichever came first. For belamaf administrations at the hospital, a gap of more than 90 days between the hospital discharge day and the next prescription defined a discontinuation. The post-belamaf period was defined as the period from belamaf treatment discontinuation to the end of follow-up or the end of the study period, death, or loss to follow-up, whichever occurred first. MM: multiple myeloma

This was a retrospective real-world study of anonymized German health insurance claims data from AOK PLUS on patients with RRMM (Fig. 1). AOK PLUS is a regional statutory health insurance fund covering approximately 3.5 million individuals in the German states of Saxony and Thuringia. The study complied with all applicable laws regarding subject privacy. No direct subject contact or primary collection of individual human subject data occurred. Study results were in tabular form and presented as aggregate analyses that omit subject identification; therefore informed consent to participate, human ethics declarations, or ethics committee or Internal Review Board approval were not applicable.

The study included adult patients treated with belamaf between August 1, 2020, and March 31, 2023 (with the first belamaf treatment as the index date), and a prior first diagnosis of MM between January 1, 2010, and the index date (if the first belamaf treatment was received in the hospital, the admission date from the hospitalization was used as index date). The claims dataset had no direct information on whether patients were relapsed or refractory (i.e., RRMM). However, since at the time of the study period belamaf was approved for treatment in fifth-line or higher, if prescribed according to the approved label, patients with RRMM were implicitly captured. Patients with one or more inpatient and/or two confirmed outpatient diagnoses of MM (International Statistical Classification of Diseases and Related Health Problems, 10th Revision, German Modification [ICD-10-GM] code C90.0) in two different quarters within 12 months between January 1, 2010, and first belamaf treatment were included. For patients with two confirmed outpatient diagnoses, the first diagnosis was defined as the diagnosis date. In addition, patients had to be continuously insured for at least 12 months prior to the index date.

The study timeframe was divided into three periods: (1) the pre-belamaf period was defined as the time from 1 month before the start of belamaf treatment to the start of belamaf treatment; (2) the belamaf treatment period started at the index date and ended on the last day of the first instance of a 90-day period without a subsequent dose, end of follow-up, or date of death, whichever came first (for belamaf administrations at the hospital, a gap of more than 90 days between the hospital discharge day and the next prescription defined a discontinuation); (3) the post-belamaf period was defined as the time from belamaf treatment discontinuation to the end of follow-up or end of study period, death, or loss to follow-up, whichever occurred first. Patients were censored if they received re-treatment in the postbelamaf period.

### Study objectives

The primary objective of this study was to describe the baseline characteristics of patients with MM treated with belamaf. Secondary objectives included describing ocular events in patients with ocular visits documented in the claims data, and reporting time-to-event outcomes of patients included in the study.

### Study assessments

Primary outcomes were investigated in a baseline period of 12 months before the first belamaf treatment (pre-belamaf period) and included sex, age at index date, co-morbidities, level of care at index date, and treatment history (prior treatments by agent) as well as ocular diagnoses (ICD-10-GM). Level of care was used as an indicator of frailty describing how impaired patients were in their daily activities (0: no impairment, 1: minor impairment, 2: significant impairment, 3: serious impairment, 4: severe impairment, 5: highest impairment). Secondary outcomes were investigated before, during, and after the belamaf treatment period and included ocular events and time-to-event outcomes. Ocular events included but were not limited to keratitis, other disorders of the cornea, glaucoma, cataract, background retinopathy and retinal vascular changes, corneal scars and opacities, visual disturbances, other affections of the lacrimal gland including dry eye, senile incipient cataract, senile cataract (unspecified), retinal hemorrhage, and visual impairment including blindness. Time-to-event outcomes included: time until first dose hold; time to treatment discontinuation (TTD); time to next treatment (TTNT); real-world progression-free survival (rwPFS); and overall survival (OS) (all-cause death). Time until first dose hold was defined as the time from the start of belamaf therapy until the first dose hold; a dose hold was defined as a period of > 28 days between belamaf administrations. Censoring occurred at belamaf treatment discontinuation, the start of a new treatment line, the end of the study period, or loss to follow-up. TTD was defined as the time from the start of belamaf therapy until its discontinuation. TTNT was defined as the time from the start of belamaf treatment until the start of the next line of therapy. rwPFS was defined as the time from the start of belamaf therapy until progression to a new treatment line or death due to any cause, whichever occurred first. OS was defined as the time from the start of belamaf therapy until death due to any cause. Censoring for TTD, TTNT, rwPFS, and OS occurred at the end of the study period or loss to follow-up.

### Statistical analysis

The study reported descriptive data, and no hypothesis testing was performed. For categorical (including dichotomous) variables, frequencies and percentages were reported along with corresponding sample sizes. Continuous variables were summarized using mean, standard deviation, median, and interquartile range (IQR) along with corresponding sample sizes. Rates were reported per patient-year and with 95% confidence intervals (CIs). For time-to-event outcomes, median time to events with 95% CIs were reported and Kaplan–Meier figures were provided. No statistical comparisons across patient groups or datasets were included in the scope of this project. Analyses were conducted in R (version 4.1.3 or higher) and STATA software version 17 (STATA Corp., College Station, Texas).

## Results

Overall, 31 patients with RRMM were included in the pre-belamaf and belamaf treatment period, while 22 of these patients were followed in the post-belamaf period. All patients were previously treated with lenalidomide, bortezomib, and dexamethasone, and 90.3% of patients had also received daratumumab, making them triple-class exposed. In the belamaf period, the median number of belamaf prescriptions per patient was 3 (range 1–14), and the median time between belamaf prescriptions was 21 days (range 7–84 days). Three patients were censored due to belamaf re-treatment after a 90-day gap in the post-belamaf period. Of the nine patients without follow-up, eight had died prior to the start of the post-belamaf period and one had continued treatment until the end of the study period.

### Baseline characteristics

The overall cohort consisted of 31 adult patients with RRMM treated with belamaf (Table 1). The median age was 66 years and 51.6% were female. The median Charlson Comorbidity Index score [12] calculated from the diagnoses observed in the 12 months pre-index was 5 (IQR 4–8). Most patients showed significant impairment based on the level of care (median: 2 [IQR 0–3]). The most common 12-month pre-index comorbidities in this patient population were renal disease (*n* = 17; 54.8%), cardiovascular disease (*n* = 15; 48.4%), and congestive heart failure (*n* = 8; 25.8%).

**Table 1:** Baseline characteristics

	RRMM cohort (*N* = 31)
Sex, *n* (%)	
Female	16 (51.6)
Male	15 (48.4)
**Age at index (years)**	
Mean (SD)	67.4 (10.5)
Median	66
Interquartile range	61–73
**Age groups**, **n (%)**	
< 65 years	14 (45.2)
65–74 years	10 (32.3)
≥ 75 years	7 (22.5)
**CCI score** ^†^	
Mean (SD)	5.8 (3.2)
Median	5
Interquartile range	4–8
**CCI score**^†^, **n (%)**	
0	0 (0)
1	0 (0)
2	5 (16.1)
3	1 (3.2)
4	7 (22.6)
≥ 5	18 (58.1)
**Level of care** ^‡^	
Mean (SD)	1.6 (1.5)
Median	2
Interquartile range	0–3
**Level of care**^‡^, **n (%)**	
0	11 (35.5)
1	3 (9.7)
2	7 (22.6)
3	7 (22.6)
4	2 (6.4)
5	1 (3.2)
**Pre-index comorbidities**, **n (%)**	
Renal disease	17 (54.8)
Cardiovascular disease	15 (48.4)
Congestive heart failure	8 (25.8)
Diabetes mellitus	7 (22.6)
Anemia	7 (22.6)
Chronic pulmonary disease	6 (19.4)
Polyneuropathy	6 (19.4)
History of myocardial infarction	2 (6.5)
Extramedullary disease	2 (6.5)
Peripheral vascular disease	1 (3.2)
Dementia	1 (3.2)
Any liver disease	1 (3.2)
Peptic ulcer disease	1 (3.2)
Rheumatologic disease	1 (3.2)
COVID-19	1 (3.2)
**Prior MM treatments**, **n (%)**	
Chemotherapy	18 (58.1)
Corticosteroids	31 (100)
Proteasome inhibitors	31 (100)
Immunomodulators	31 (100)
Monoclonal CD38 antibodies	29 (93.5)
Histone deacetylase	2 (6.5)

^†^CCI was calculated within the 12-month baseline period prior to index; ^‡^Level of care (“Pflegestufe”) was provided as an indicator of frailty describing how impaired patients were in their daily activities. 0: no impairment, 1: minor impairment, 2: significant impairment, 3: serious impairment, 4: severe impairment, 5: highest impairment

CCI: Charlson Comorbidity Index; COVID-19: coronavirus disease 2019; MM, multiple myeloma; RRMM: relapsed/refractory multiple myeloma; SD: standard deviation

All patients in the study cohort had received several MM therapies prior to treatment with belamaf (Table 1). All patients had received corticosteroids, proteasome inhibitors, and immunomodulators. Most patients had received treatment with monoclonal CD38 antibodies (93.5%) and/or chemotherapy (58.1%).

### Ocular events

Of the 31 patients included in the pre-belamaf and belamaf treatment periods, 10 (32.3%) and 16 (51.6%) patients had one or more recorded ophthalmological visit, respectively. Of the 22 patients in the post-belamaf treatment period, 15 (68.2%) had one or more recorded ophthalmological visit after treatment discontinuation.

### Prevalent ocular diagnoses and ocular events of interest

Table 2 reports the frequency of prevalent ocular diagnoses stratified by study periods. In the pre-belamaf period, 11 (35.5%) patients had at least one prevalent ocular diagnosis record. In the belamaf treatment period, 21 (67.7%) patients had at least one ocular diagnosis record. In the post-belamaf period, 18 (81.1%) patients had at least one ocular diagnosis record after treatment discontinuation.

**Table 2:** Frequency of prevalent ocular diagnoses stratified by study periods

Most frequent ocular diagnoses, *n* (%)	Pre-belamaf period (*n* = 31)	Belamaf period (*n* = 31)	Post-belamaf period (*n* = 22)
Glaucoma suspect (H40.0)	3 (9.7)	3 (9.7)	–
Other disorders of lacrimal gland (H04.1)	2 (6.5)	2 (6.5)	3 (13.6)
Background retinopathy and retinal vascular changes (H35.0)	2 (6.5)	3 (9.7)	–
Senile cataract, unspecified (H25.9)	1 (3.2)	2 (6.5)	–
Keratoconjunctivitis (H16.2)	1 (3.2)	–	–
Age-related incipient cataract (H25.0)	1 (3.2)	–	3 (13.6)
Unspecified cataract (H26.9)	–	3 (9.7)	2 (9.1)
Other and unspecified superficial keratitis without conjunctivitis (H16.1)	–	3 (9.7)	3 (13.6)
Unspecified acute conjunctivitis (H10.3)	–	–	2 (9.1)
Unspecified visual disturbance (H53.9)	–	–	2 (9.1)
Other refractive diagnoses	15 (48.4)	27 (87.1)	21 (95.5)

The pre-belamaf period was defined as the time from 1 month before the start of belamaf treatment to the start of belamaf treatment. The belamaf period started at the index date and ended on the last day of the first instance of a 90-day period without a subsequent dose, the end of follow-up, or the date of death, whichever came first. For belamaf administrations at the hospital, a gap of more than 90 days between the hospital discharge day and the next prescription defined a discontinuation. The post-belamaf period was defined as the period from belamaf treatment discontinuation to the end of follow-up. Other refractive diagnoses included presbyopia (H52.4), hypermetropia (H52.0), astigmatism (H52.2), and myopia (H52.1)

The number and frequency of documented prevalent ocular events of interest stratified by study periods are shown in Table 3. Keratitis was the most frequent ocular diagnosis across all three study periods.

**Table 3:** Prevalent ocular events of interest stratified by Belamaf treatment period

Ocular diagnoses of interest, *n* (%)	Pre-belamaf period (*n* = 31)	Belamaf period (*n* = 31)	Post-belamaf period (*n* = 22)
Keratitis (H16)	3 (9.7)	6 (19.4)	6 (27.3)
Other disorders of the cornea (H18)	0 (0)	3 (9.7)	3 (13.6)
Glaucoma (H40.42)	3 (9.7)	3 (9.7)	2 (9.1)
Cataract, unspecified (H26.9)	0 (0)	3 (9.7)	2 (9.1)
Background retinopathy and retinal vascular changes (H35.0)	2 (6.5)	3 (9.7)	2 (9.1)
Corneal scars and opacities (H17)	0 (0)	2 (6.5)	2 (9.1)
Visual disturbances (H53)	1 (3.2)	2 (6.5)	2 (9.1)
Other affections of the lacrimal gland, including dry eye (H04.1)	2 (6.5)	2 (6.5)	3 (13.6)
Senile incipient cataract (H25.0)	1 (3.2)	2 (6.5)	3 (13.6)
Senile cataract, unspecified (H25.9)	1 (3.2)	2 (6.5)	2 (9.1)
Retinal hemorrhage (H35.6)	0 (0)	1 (3.2)	0 (0)
Visual impairment including blindness (H54)	1 (3.2)	1 (3.2)	1 (4.5)

The pre-belamaf period was defined as the time from 1 month before the start of belamaf treatment to the start of belamaf treatment. The belamaf period started at the index date and ended on the last day of the first instance of a 90-day period without a subsequent dose, the end of follow-up, or the date of death, whichever came first. For belamaf administrations at the hospital, a gap of more than 90 days between the hospital discharge day and the next prescription defined a discontinuation. The post-belamaf period was defined as the period from belamaf treatment discontinuation to the end of follow-up

### Incident ocular events

**Fig. 2:**
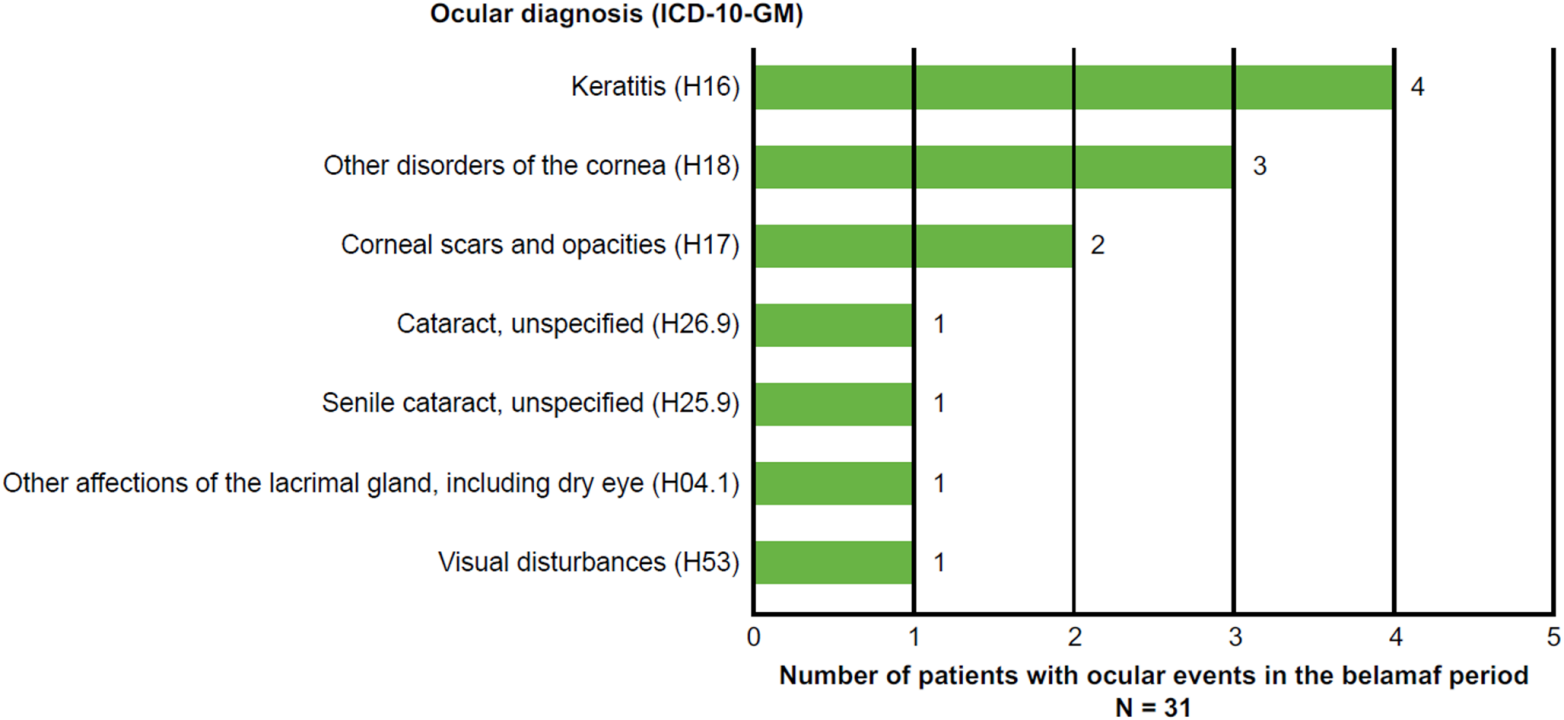
Incident ocular events in the belamaf period. ICD-10-GM: International Statistical Classification of Diseases and Related Health Problems, 10th Revision, German Modification

To capture incident events, only newly diagnosed events per patient were also assessed, considering previous ocular diagnoses within 12 months pre-index (Fig. 2). Seven (22.6%) patients had ≥ 1 incident diagnosis of interest during the belamaf treatment period. The most frequent ocular diagnoses were corneal-related events.

### Time-to-event outcomes

**Fig. 3:**
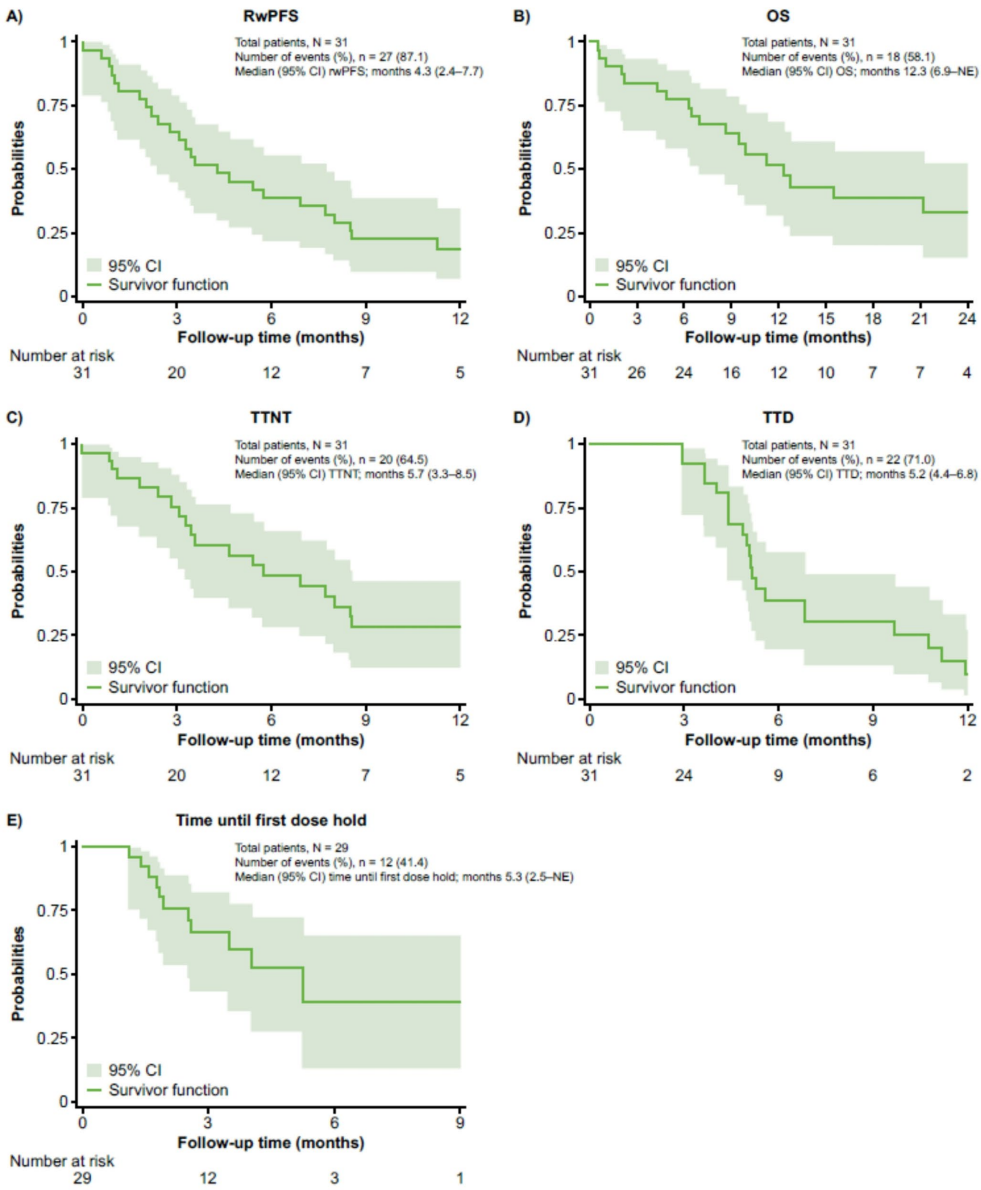
Time-to-event outcomes. rwPFS: Belamaf treatment start to the start of the next treatment line or death (censoring at the end of the study period or loss to follow-up). OS: Belamaf treatment start to death (censoring at the end of the study period or loss to follow-up). TTNT: belamaf treatment start to the start of the next treatment line (censoring at the end of the study period, loss to follow-up, or death). TTD: belamaf treatment start to discontinuation (censoring at the end of the study period, loss to follow-up, or death). Time until first dose hold: A dose hold is defined as a period of > 28 days between belamaf administrations; censoring occurred at belamaf treatment discontinuation, the start of a new treatment line, the end of the study period, or loss to follow-up (with death defined as a competing event). CI: confidence interval; NE: not evaluable; OS: overall survival; rwPFS: real-world progression-free survival; TTD: time to treatment discontinuation; TTNT: time to next treatment

The median time for rwPFS was 4.3 months (95% CI 2.4–7.7) (Fig. 3A). The event-free probability was 64.5% (95% CI 45.1–78.5) at 3 months and 38.7% (95% CI 22.0–55.1) at 6 months. The median OS was 12.3 months (95% CI 6.9–not evaluable [NE]) (Fig. 3B); 58.1% of patients had died by the time of data cutoff. The survival probability was 83.9% (95% CI 65.5–92.9) at 3 months, 77.4% (95% CI 58.4–88.5) at 6 months, and 51.6% (95% CI 31.8–68.2) at 12 months.

The median TTNT, TTD, and time until first dose hold were 5.7 months (95% CI 3.3–8.5), 5.2 months (95% CI 4.4–6.8), and 5.3 months (95% CI 2.5–NE), respectively (Fig. 3C–E).

## Discussion

To our knowledge, this is the first real-world study of baseline characteristics, ocular events, and clinical outcomes of patients with RRMM treated with belamaf, specifically in Germany. We report a descriptive analysis based on anonymized claims data from the regional German statutory health insurance fund AOK PLUS. Patients had received multiple prior treatments, including proteasome inhibitors, immunomodulators, and monoclonal CD38 antibodies, indicating our study consisted of patients with relapsed or refractory disease. Of note, two patients had not received prior treatment with monoclonal CD38 antibodies, as specified in the original European label for belamaf. A significant health impairment status at baseline was observed prior to treatment administration and there was a high proportion of severe comorbidities, with approximately half of the patients having renal and/or cardiovascular diseases and one-quarter having congestive heart failure.

According to the original European label, ophthalmic consultations were a main part of the recommended supportive care in the treatment with belamaf; ophthalmic examinations should be performed by an eye care professional at baseline and before each subsequent three treatment cycles, and as clinically indicated while on treatment [9]. The activity recorded within a claims dataset is directly related to the financial invoicing of the clinicians. Ophthalmic activity was significantly below the label recommendation, suggesting either the absence of the recommended activity or underreporting of consultations (e.g., clinicians not reporting all consultations within the same quarter due to reimbursement reasons).

In line with our findings, real-world studies and clinical trials reported belamaf treatment was well tolerated [6–8, 13, 14]. However, the proportion of patients with ocular events in our study appears to be lower than reported in the literature for both belamaf monotherapy and combination therapy [6–8, 13, 14]. In the DREAMM-2 trial, 74% of patients receiving belamaf 2.5 mg/kg once every 3 weeks experienced ocular adverse events including keratopathy (71%), blurred vision (25%), and dry eye (18%) [6]. In the DREAMM-7 trial, 79% of patients receiving belamaf in combination with Vd experienced ocular adverse events including blurred vision (66%) and dry eye (51%) [7]. In the DREAMM-8 trial, 79% and 61% of patients receiving belamaf in combination with Pd experienced blurred vision and dry eye, respectively [8]. In a US observational study of 30 adult patients with MM treated with belamaf monotherapy at the Duke Cancer Institute between 2020 and 2022, 53%, 17%, and 14% of patients experienced blurry vision, dry eye, and photophobia, respectively [13]. In another US electronic health record–derived study, the Flatiron Health Database observational study, of 137 adult patients with MM treated with belamaf, 40.9%, 32.1%, 19.7%, and 12.4% experienced keratopathy, blurred vision, dry eye, and keratitis, respectively [15]. In our study, nine (29%) patients experienced keratitis and other disorders of the cornea, two (6.5%) patients experienced visual disturbances, and two (6.5%) patients had other affections of the lacrimal gland, including dry eye, during the belamaf treatment period.

Our study demonstrated that there was a lower number of documented incident ocular events than anticipated (*n* = 7; 22.6%). The increased number of reported ocular events in the DREAMM-2, DREAMM-7, and DREAMM-8 clinical trials compared with our study may be attributed to the high levels of monitoring, including protocol-mandated eye examinations and solicitation of ocular symptoms. The decreased number of reported ocular events in comparison to US observation studies is hard to attribute without specific exploration but could be a result of differences in US vs. German clinical practice, reimbursement data collection, and patient susceptibility. Regardless of cause(s), this study reinforces findings that a proportion of the ocular symptoms predicted by DREAMM trials will be transparent to health insurers.

Time-to-event outcomes were consistent with the literature. Our study reports a median rwPFS of 4.3 months (95% CI 2.4–7.7) in patients with RRMM treated with belamaf monotherapy; real-world studies have previously reported a rwPFS time of 2–5.4 months [14–16]. Our study reports a median OS of 12.3 months (95% CI 6.9–NE), which is slightly higher than in observational reports; studies in patients with belamaf reported a median OS between 6.5 and 7.9 months [14, 16]. In the final analysis of the DREAMM-2 trial, patients who were treated with belamaf 2.5 mg/kg every 3 weeks had a median PFS of 2.8 months and a median OS of 15.3 months [6]. Based on our results and the current literature, RRMM remains a disease with a poor prognosis.

Our study had a number of limitations. This study uses claims data, which as a financial tool represents clinical activity and diagnosis relevant for reimbursement and invoiced for; clinical activities and clinical details may therefore not exactly mirror patient or clinician experience based on reimbursement rules or data process and invoicing practices. Similarly, while insurance claims data can track prescriptions approved for reimbursement, information on actual medication use and patient adherence was not available. Despite this, coding in claims databases is considered of high quality and all relevant cancer treatments are typically reimbursed in Germany. Patient information on prior lines of therapy was not available in the claims data. However, according to the European label, we assumed that patients undergoing belamaf treatment were relapsed or refractory. Based on available patient data, it was estimated that 90.3% of all patients were triple-class exposed. Additionally, disease progression was not recorded in the database. Therefore, rwPFS was used as a proxy measure, a common approach in real-world evidence studies, based on progression to a new treatment line or death. Furthermore, our study focuses on ocular adverse events; MM and the use of belamaf can cause additional non-ocular adverse events that are not discussed here. The claims data do not report information on worsening or resolution of specific ocular conditions, and therefore incident events were defined as only newly diagnosed events per patient while also considering any previous ocular diagnoses within 12 months before the index date. Due to the descriptive nature of this study, confounding factors including concomitant medications, socioeconomic patient background, and level of support, which may have contributed to observed events and timings, were not controlled. The comparisons of the number of patients who experienced ocular events may be impacted using Medical Dictionary for Regulatory Activities Preferred Term coding in clinical trials, as opposed to ICD-10-GM codes in this study. Lastly, the study findings are limited by the small number of patients with RRMM, variability in disease duration and follow-up periods, and the differing number of patients across each study period and follow-up length in the time-to-event outcomes.

## Conclusions

Our findings highlight the importance of ocular supportive care during belamaf treatment in real-world clinical practice. Patients with RRMM were frail and had severe comorbidities prior to belamaf treatment initiation. Compared with the DREAMM-2, DREAMM-7, and DREAMM-8 clinical trials and observational studies, the number of reported ocular events in this study appeared to be lower. A tolerable ocular toxicity profile was observed with belamaf treatment. Overall, RRMM remains an aggressive disease with a poor prognosis.

## Data Availability

Data used for this publication were generated by AOK PLUS. For access to anonymized subject-level data, please contact AOK PLUS.
